# Development of In Vitro 3D TissueFlex® Islet Model for Diabetic Drug Efficacy Testing

**DOI:** 10.1371/journal.pone.0072612

**Published:** 2013-08-15

**Authors:** Zhaohui Li, He Sun, Jianbin Zhang, Haijiao Zhang, Fanyu Meng, Zhanfeng Cui

**Affiliations:** 1 Institute of Biomedical Engineering, University of Oxford, Oxford, United Kingdom; 2 Tianjin Weikai Bioeng Ltd, Tianjin, China; Broad Institute of Harvard and MIT, United States of America

## Abstract

Increasing individuals diagnosed with type II diabetes pose a strong demand for the development of more effective anti-diabetic drugs. However, expensive, ethically controversial animal-based screening for anti-diabetic compounds is not always predictive of the human response. The use of *in vitro* cell-based models in research presents obviously ethical and cost advantages over *in vivo* models. This study was to develop an *in vitro* three-dimensional (3D) perfused culture model of islets (Islet TF) for maintaining viability and functionality longer for diabetic drug efficacy tests. Briefly fresh isolated rat islets were encapsulated in ultrapure alginate and the encapsulated islets were cultured in TissueFlex^®^, a multiple, parallel perfused microbioreactor system for 7 days. The encapsulated islets cultured statically in cell culture plates (3D static) and islets cultured in suspension (2D) were used as the comparisons. In this study we demonstrate for the first time that Islet TF model can maintain the *in vitro* islet viability, and more importantly, the elevated functionality in terms of insulin release and dynamic responses over a 7-day culture period. The Islet TF displays a high sensitivity in responding to drugs and drug dosages over conventional 2D and 3D static models. Actual drug administration in clinics could be simulated using the developed Islet TF model, and the patterns of insulin release response to the tested drugs were in agreement with the data obtained *in vivo*. Islet TF could be a more predictive *in vitro* model for routine short- and long-term anti-diabetic drug efficacy testing.

## Introduction

The number of individuals diagnosed with type II diabetes, which is caused by the ‘metabolic syndrome’ – obesity, insulin resistance and/or abnormal insulin secretion, is increasing worldwide, and creating a strong demand for the development of more effective anti-diabetic drugs [1]. However, animal-based screening for anti-diabetic compounds requires sacrifice of a large number of animals, which is expensive, ethically controversial, and not always predictive of the human response [2,3]. The use of *in vitro* cell-based models in research presents obviously ethical and cost advantages over *in vivo* models. Traditionally, *in vitro* research has been conducted using 2D cell cultures. However, conventional 2D cell culture where cells are cultured on flat, rigid plastic substrates does not reproduce the tissue architecture *in vivo*, and do not forecast organ-specific toxicity [4,5]. This is because real tissues have a 3D geometry, gel-like stiffness, and complex organisation of extracellular matrix (ECM). 3D cell cultures establish cell-cell contacts and cell-ECM interactions by embedding cells in 3D scaffolds that mimics the biochemistry and mechanics of the microenvironment *in vivo*, hence offers a practical alternative to natural tissue models.

Pancreatic islets of Langerhans are highly metabolically active mini-organelles that secrete hormones to regulate blood glucose. Therefore islets are a key focus of diabetes research and diabetic drug efficacy testing. However, isolated islets rapidly lost mass, viability and functions during *in vitro* culture [6]. There are several reasons and complexities(1). Unlike many single cell types, islets do not proliferate in culture; hence there may be mass loss of islet in culture [7]. (2) Due to the highly metabolically feature of islets and the size of the islets, the central core can become necrotic, probably as a result of inadequate oxygen supply [8], resulting in loss of viability. (3) The destruction of the islet microenvironment and the loss of basement membrane support that occur during enzymatic isolation and purification lead to a cellular stress to islets that impair their function and survival [9]. (4) The conventional suspension tissue culture of islets after isolation could also cause the loss of islet viability. Islets tend to aggregate in suspension culture which often causes central necrosis of these large cellular aggregates [6]. As a result, functional islets cannot be cultured for longer period of time, which is a bottleneck for testing chronic and accumulative drug effect where long term culture is essential. Therefore developing simple and cost-effective *in vitro* models which maintain long-term islet viability and functionalities is vital for study of diabetes and anti-diabetic drug efficacy testing.

Extracellular matrix is one of the most important components of the islet microenvironment and plays a significant role inducing islet growth and differentiation [10]. Therefore selection of scaffold materials which mimic the microenvironment of islets *in vivo* is of vital importance. Although natural ECM such as Type I collagen and Matrigel are widely used as scaffolds to improve *in vitro* cell survival and function, their intrinsic properties such as biochemically complexity, inconsistence in quality and ill-defined sources limit their applications and introduce further complexity in interpretation of the data [11]. By contrast natural-derived and synthetic hydrogels are particular useful because they have a high water content to promote cell proliferation and are structurally and mechanically similar to the native ECM of many of soft tissues [12]. For instance, alginate, a naturally derived polysaccharide extracted from seaweed alga, has been extensively used as a hydrogel ECM for cell immobilization, cell transplantation, and tissue engineering [6,13]. The cell immobilization procedure using alginate can be carried out in a single-step process under very mild conditions, and is therefore compatible with most living cells [14]. Alginate is regarded as inert and hence does not interfere with cellular biochemical interactions apart from providing a mechanical support. Another outstanding advantage in using alginate is the relative simplicity in recovering the encapsulated cells.

Culture conditions have a profound effect on the cell viability and function in 3D scaffolds [15,16]. For instance, perfusion culture of hepatocytes retained liver metabolic functions comparable to those *in vivo* [17]. In the body, islets are well perfused through fenestrated capillary endothelial cell lining, which is essential for the supply of oxygen and nutrients to the cells in their inner core [18]. For instance, islets receive 5 to 15% of their total blood flow of the pancreas even though they constitute about 1% of the pancreas by weight [19]. As the vasculature was disrupted during the process of islet isolation, the cells in the inner core of the islets could not receive an adequate supply of oxygen and nutrients during culture, as diffusion is the only way for oxygen and nutrient supply in static culture *in vitro* [20]. Although rapid revascularization at various level post-transplantation has been observed [18], the ability of microvasculature regeneration was complete lost when islets were cultured *in vitro*. Therefore providing a blood flow-like system for *in vitro* islet culture, particularly in 3D encapsulation to mimic the vascularization *in vivo* is the key for successful development of an *in vitro* functional islet model.

Here we developed an *in vitro* 3D perfused islet model (Islet TF) for diabetic drug study. Fresh isolated rat islets were encapsulated in ultrapure alginate beads and the encapsulated islets were cultured in TissueFlex^®^ (Zyoxel Ltd, UK), a multiple, parallel perfused microbioreactor system, purposely designed for 3D culture [21]. The encapsulated islets statically cultured in cell-culture plates (3D static) and standard islet suspension culture (2D) were also tested for comparisons. Islets were cultured for 7 days under different culture conditions stated above. Islet morphology, viability, and functional insulin release response to glucose stimulation were subsequently examined to assess various culture models. As a demonstration case study for diabetic drug efficacy testing, two diabetic drugs tolbutamide and GLP-1 were tested using the developed Islet TF model with defined concentrations at various test conditions. The sensitivity of *in vitro* Islet TF model response to drugs was assessed by determining of dynamic insulin release into the culture medium, and the results compared with those obtained in 3D static culture and standard 2D culture conditions.

## Methods

### Islet isolation

The experimental work was approved by the Committee on the Ethics of Animal Experiments of Tianjin Weikai Bioeng Ltd, following the guidelines used in the UK, and the Committee on the Ethics of Animal Experiments of Tianjin International Joint Academy for Biotechnology and Medicine (TJAB) where this part of experiment was carried out. Islet isolation was carried out strictly following the established procedure [22] with every effort to minimize the animal suffering. Briefly, under chloral hydrate anaesthesia, the abdomen of fed male Wistar rats (250 g body weight) was opened and the common bile duct was cannulated. The pancreas was distended by infusion of 10 ml sterile collagenase Hanks’ balanced salt solution (type V, 1 mg/ml; Sigma). The pancreas was then removed and placed into a 50 ml Falcon tube containing collagenase solution and enzymatically digested in a water bath at 37^°^ C for 20 min by occasionally shaking. The digestion process was stopped by the addition of cold HBSS containing 5% FBS. The digested tissue was then passed through a mesh filter with a pore size of 80 µm. The filtrate was washed with cold HBSS by centrifugation (800 rpm, 10 min, 4 ^°^C). The islet pellets were resuspended in 5 ml DMEM-F12 (31331-028, Invitrogen) containing 5.5 mM D-glucose supplemented with 2% human albumin, 10 mM HEPES and 100 U/ml penicillin, 100 µg/ml streptomycin (complete culture medium) and purified by hand-picking under a microscope. In average about 120 islets with diameter of 80-100 µm was obtained per rat. Pooled rat islets from two or three rats were used in each experiment. Purified pooled islets were split to three groups for two-dimensional standard suspension culture (2D), three-dimensional static culture (3D static) and 3D perfused TissueFlex^®^ culture (Islet TF).

### 3D encapsulation of islets

After isolation, two groups of islets termed as 3D static and Islet TF cultures were resuspended in complete culture medium and mixed with 6% (w/v) highly purified, high guluronic content alginate (MVG, NovaMatrix) to form final 1.5% (w/v) cell-alginate solution. Alginate beads (average 900 µm in diameter) were formed by manually dripping the cell-alginate solution through a 25 gauge needle into a solution containing 100 mM Ca^2+^ ions. Alginate beads were then washed once with 0.9% NaCl and twice with complete culture medium.

### 2D and 3D culture of islets

For 2D culture, 10 islets were transferred into each 35 mm of non-treated Petri dish with 1.6 ml complete culture medium and cultured at a 37 ^°^C in 5% CO_2_ incubator for 7 days. Media were changed at Day 1, 3, 5 respectively. For 3D static culture, 2 beads (containing around 10 islets) were placed in each well of a 24-well plate with 1.6 ml of complete culture medium, and cultured at a 37 ^°^C in 5% CO_2_ incubator for 7 days. Media were changed at Day 1, 3, 5 respectively. For Islet TF culture, TissueFlex^®^ perfused microbioreactor system (Zyoxel, UK) was employed. The system contains a 10-channel syringe pump, a special designed bench-top incubator and a disposable 10-well microbioreactor, which was described previously [21]. Two beads (containing around 10 islets) were transferred into each well of a 10-well microbioreactor. Ten ml of complete culture medium in each channel was supplied by the syringe pump at perfusion rate of 25 µl/h. The microbioreactor system was housed in the incubator at a 37 ^°^C with 100% humidity. Beads from three groups were taken at day 1, 3, and 7, and cell viability and glucose challenge assay were determined respectively.

### Live/dead stain

Cell viability and morphology were monitored using live/dead viability/cytotoxicity kit (L3224, Invitrogen). Briefly islets and islet-alginate beads were incubated with PBS containing 4 µM calcein and ethidium homodimer in dark at 37° C for 10 min. Samples were then washed with PBS prior to mounted on slides with mounting medium and visualized under an inverted fluorescence microscope.

### Glucose challenge assay

Glucose dependent insulin release was assayed at each time point by incubating samples in RPMI 1640 media (no glucose, 11879-020, Invitrogen) supplemented with seven serial glucose concentrations 2 mM, 4 mM, 6 mM, 8 mM, 10 mM, 12 mM and 16 mM. Briefly, samples were pre-cultured in RPMI 1640 medium containing 2 mM glucose (basal medium) for 30 min. After incubation, culture medium was removed and samples were then incubated with RPMI 1640 media containing different concentration of glucose (including 2 mM basal medium) for 15 min and then collect supernatant for insulin release assay using a commercial ELISA Kit (10-1250-01, Mercodia). Results were expressed as ng insulin release per mg cellular protein per min.

### Drug testing

Tolbutamide and GLP-1 were purchased from Sigma. Stock solution of 80 mM Tolbutamide and 200 µg/ml GLP-1 were prepared in dimethyl sulfoxide (DMSO), and aliquot and stored at -20^o^C. The working drug solution (one thousand dilutions in RPMI 1640 medium containing 8 mM glucose) was prepared depending on experimental plan before each experiment. RPMI 1640 medium containing 8 mM glucose was set as control.

### Data analysis

Data were presented as the mean ± the standard error of the mean (SEM) of three independent experiments (n = 3), each performed in duplicate. A two-tailed Student’s t-test was used with *p < 0.05; **p < 0.001 being regarded as statistically significant difference.

## Results

### Islet TF model development

**Figure 1:**
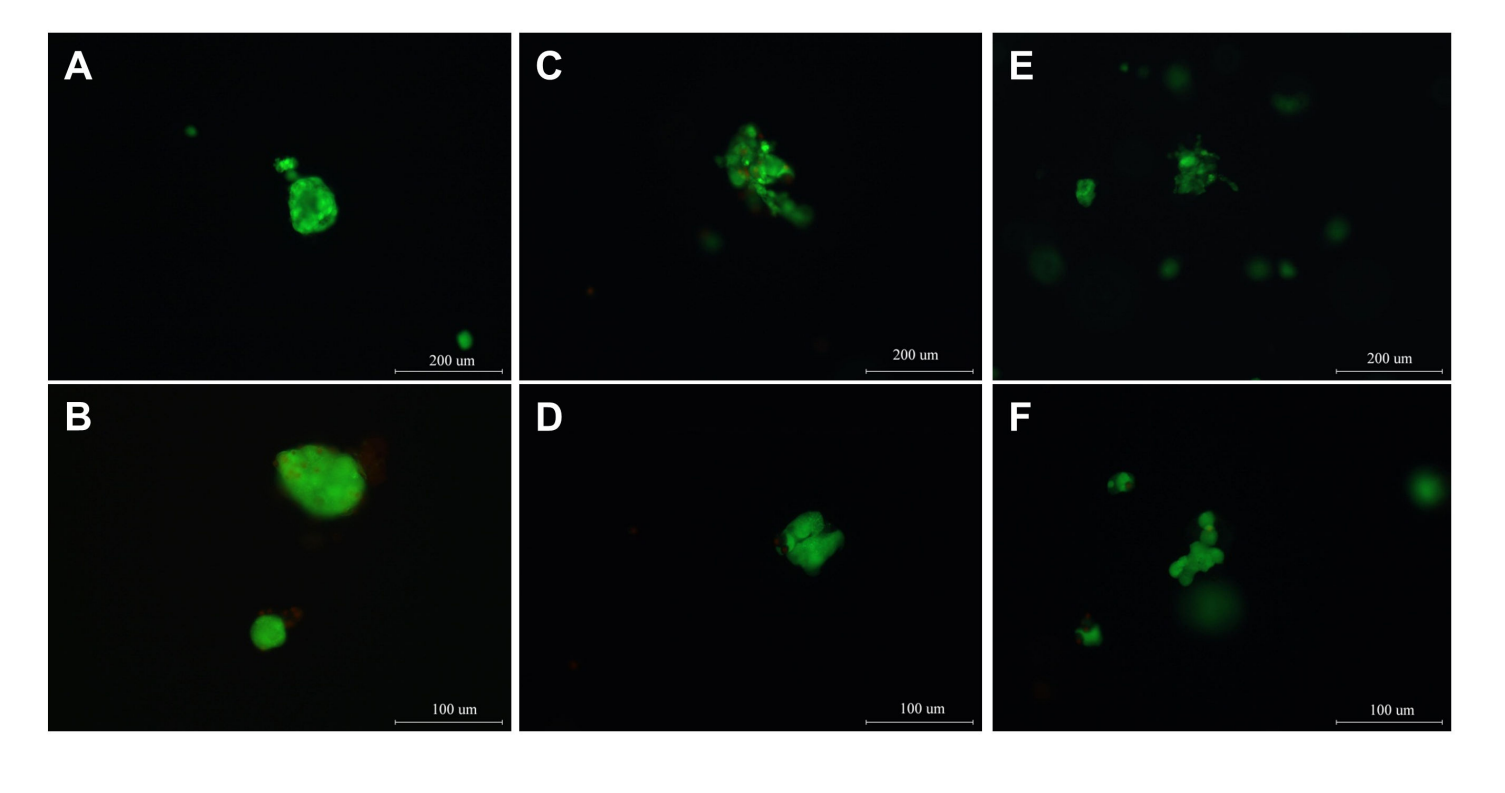
Morphology and viability of islets cultured different conditions. Morphology and viability of islets cultured in standard 2D suspension (1-A), 3D static (1-C) and Islet TF model (1-E) were assayed at Day 1 and Day7 (1-B), (1-D) and (1-F) respectively (Live islet- green, dead islets – red). Bars: A, C & E, 200µm; B, D & F, 100 µm.

**Figure 2:**
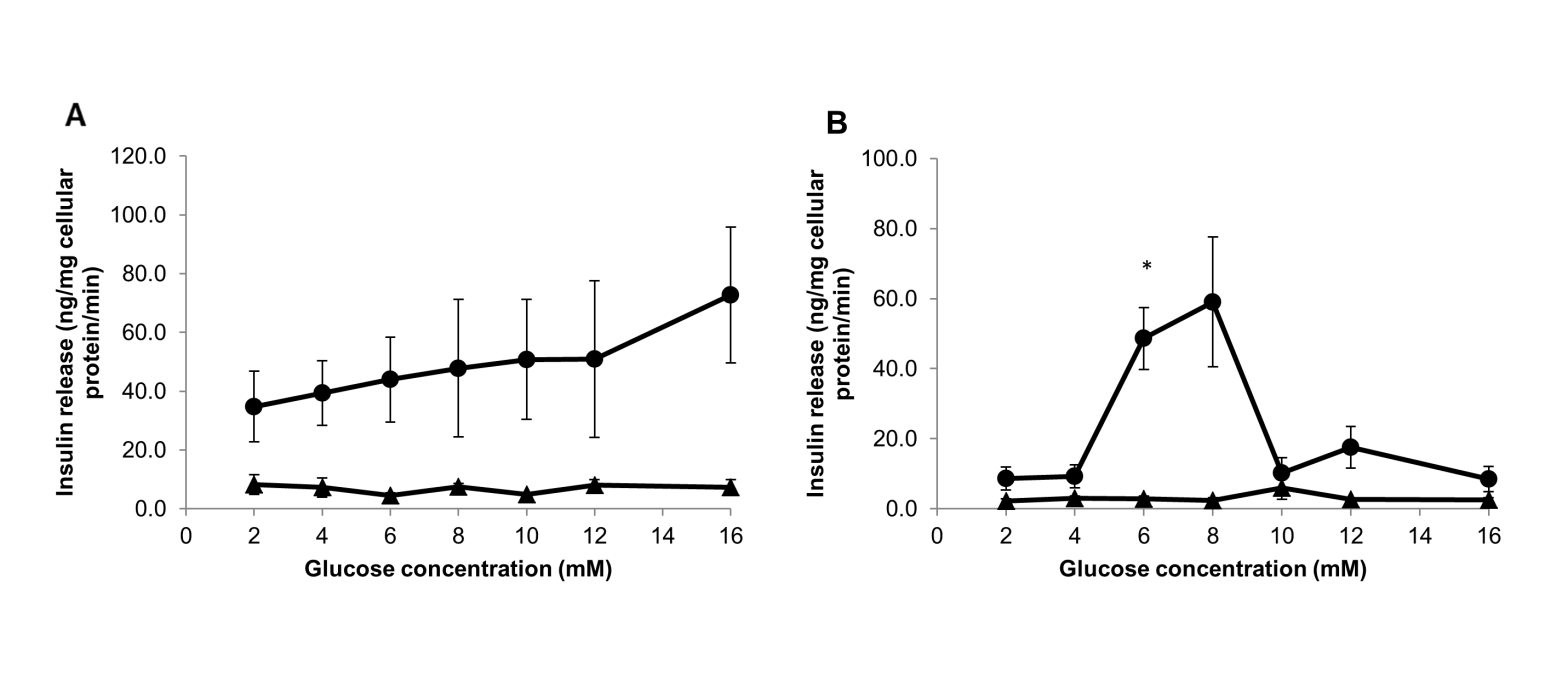
Glucose-stimulated insulin release detected from different islet models. Functional glucose-stimulated insulin release from rat pancreatic islets cultured in standard 2D (triangle) and Islet TF (circle) was detected at day 1 (A) and day 3 (B). Values are means ± SEM of 3 independent experiments, each performed in duplicate. Statistical significance of differences between Islet TF and standard 2D was calculated by Student’s T-test, * p<0.05.

After isolation, rat islets were encapsulated in highly purified MVG alginate beads (3D) and cultured under static or perfused conditions for 7 days. Islet cultured in standard suspension in petri dish (2D) was set as control. Morphology and viability of islets cultured in all three conditions were assayed using a fluorescent-based stain kit to assess any significant difference in outcome. The functional marker, insulin release response to serial concentrations of glucose, was determined using a commercial ELISA kit to evaluate the key function of islet retained during the culture period. Fluorescent images demonstrated that rat islets cultured in 3D alginate beads under both static and perfusion culture conditions maintained their intact body shape and viability in 7-day culture (Figure 1 C, D, E & F). Good islet morphology and viability were also visualized from standard 2D culture in 7-day culture (Figure 1 A & B). Although rat islets showed highly viable at Day 1 for all culture conditions (Figure 1 A, C and E), the function of islets retained in various cultures was significantly different. The functional insulin release rate from Islet TF, 3D static and standard 2D culture was determined after culturing of islets in medium containing a serial of glucose concentrations during a 15-min static incubation assay. Only did islet TF sensitively respond the concentration of glucose with a dose-dependent manner in one-day and three-day cultures. On day 1 islet TF released two folds more insulin at highest glucose (16 mM) than at lowest glucose (2 mM) whereas only little difference obtained from 3D static and standard 2D culture to response to low and high glucose concentration challenge. When culturing at 4mM glucose concentration, insulin release rate from Islet TF (39.3±11.0) was 5-fold greater than that from standard 2D culture (7.2±3.3). More significant difference was seen from islets cultured in 16 mM glucose concentration. Islet TF released over 9-fold more insulin at high glucose (72.7±23.0) compared to standard 2D culture (7.4±2.5) (Figure 2A). Interestingly after 3-day culture, the pattern of insulin release in response of glucose challenge was changed to a dose-dependent monophasic manner for both Islet TF and standard 2D models (Figure 2B). The maximal insulin release rate detected from Islet TF was 59.0±18.5 ng/mg cellular protein/min at 8 mM glucose concentration whereas the insulin release from standard 2D peaked at 10 mM glucose concentration (5.9±3.3). It was 10-fold greater of maximum insulin release from Islet TF than that from standard 2D. This was consistent with the viability data and demonstrated that Islet TF model provided a more physiological culture environment to islets and hence retained the islet viability and functions to a greater level and over a much wider range of parameters compared to 3D static and standard 2D cultures. Therefore Islet TF was used for following drug testing.

Dose response of GLP-1 and tolbutamide stimulated insulin release in Islet TF, 3D static and standard 2D culture

**Figure 3:**
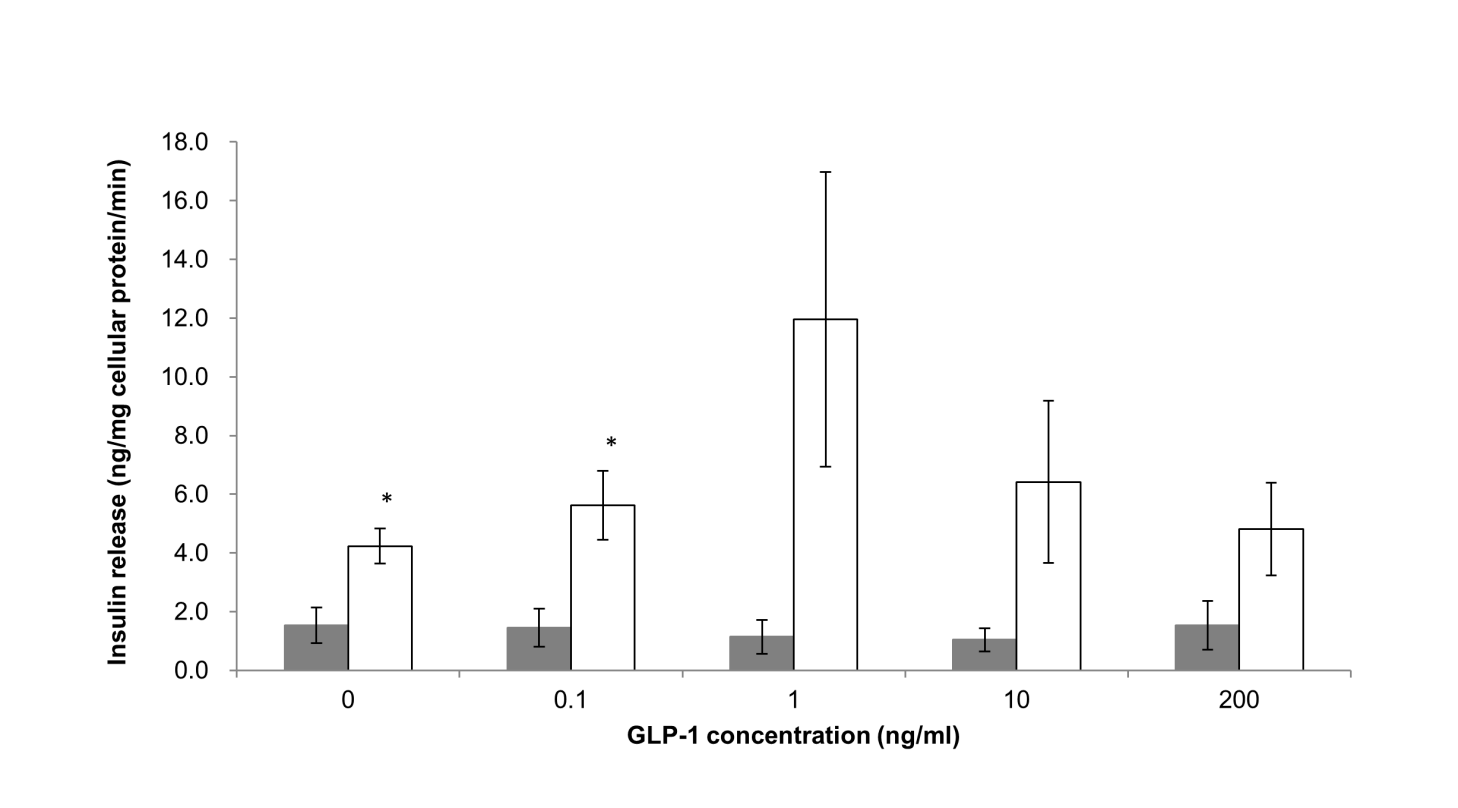
Dose response of GLP-1-stimulated insulin release detected from different islet models. Dose response of GLP-1-stimulated insulin release in standard 2D (bar in grey) and 3D static models (bar in white) in the presence of 8 mM glucose was detected. Values are means ± SEM of 3 independent experiments, each performed in duplicate. Statistical significance of differences between 3D static and standard 2D was calculated by Student’s T-test, * p<0.05.

**Figure 4:**
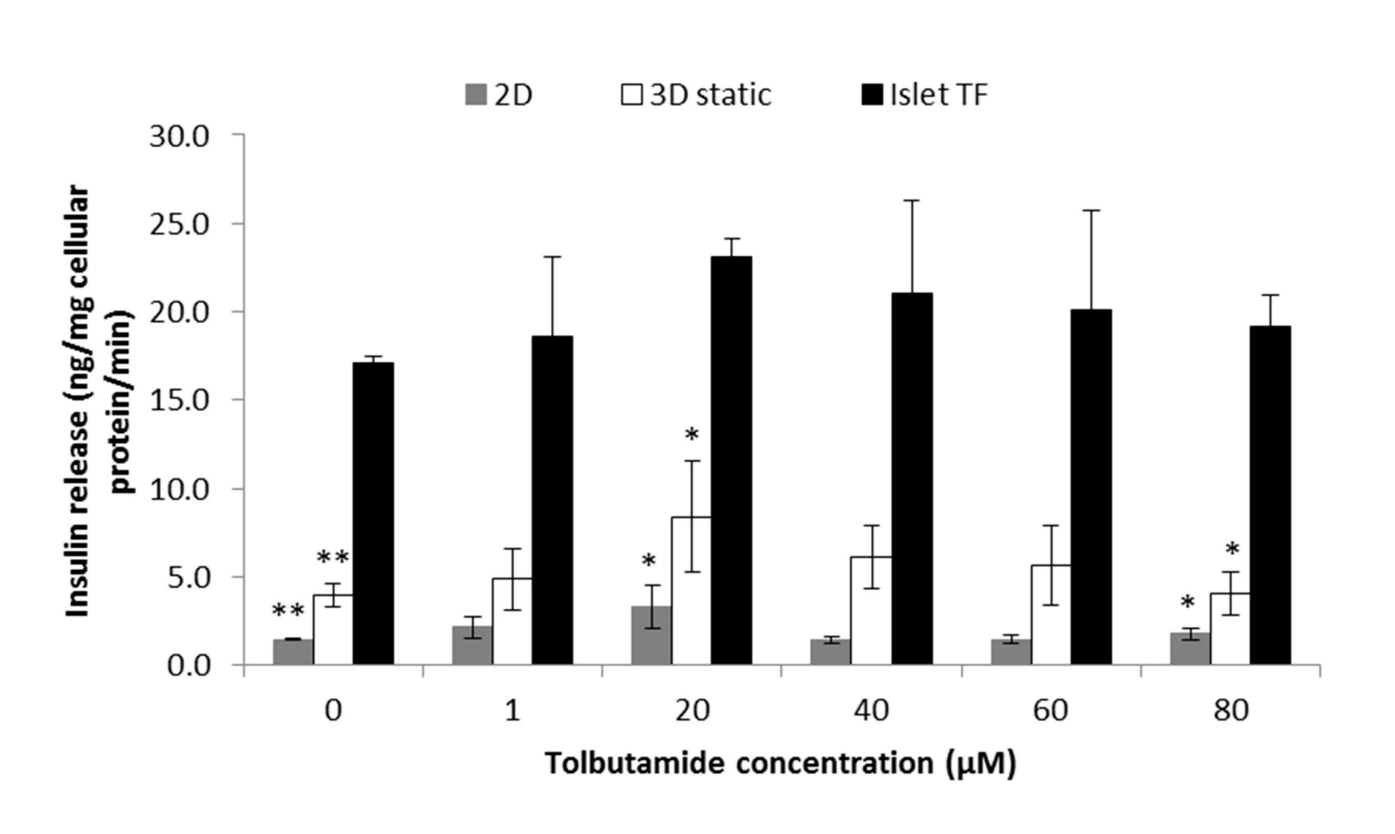
Dose response of tolbutamide-stimulated insulin release **detected from different islet models**. Dose response of tolbutamide-stimulated insulin release in standard 2D (bar in grey), 3D static (bar in white) and Islet TF models (bar in black) in the presence of 8 mM glucose was detected. Values are means ± SEM of 3 independent experiments, each performed in duplicate. Statistical significance of differences between Islet TF and standard 2D, Islet TF and 3D static models was calculated by Student’s T-test, * p<0.05 and ** p<0.001.

GLP-1 and tolbutamide were selected as model drugs to evaluate sensitivity of *in vitro* islet model developed to drugs. The dose range of GLP-1 and tolbutamide were from 0.1 to 200 ng/ml and 1 to 80 µM respectively, following the physiological dose range [23,24]. Islets cultured in 3D static and standard 2D models for GLP-1 and three models for tolbutamide were exposed in drugs with various concentrations in the presence of 8 mM glucose for 1 hour. Supernatant was then collected for insulin release assay. As shown in Figure 3, GLP-1 stimulated the insulin secretion in a dose-dependent monophasic manner observed in 3D static, and the maximal insulin release was obtained at concentration of 1 ng/ml. By contrast, no clear dose response to GLP-1 in standard 2D model and the highest insulin release was shift to concentration of 200 ng/ml. The maximum drug-induced insulin release from 3D static was 7 times higher than from standard 2D model (11.96±5.02 ng insulin/mg cellular protein/min tested from 3D static at 1 ng/ml GLP-1 whereas 1.53±0.83 was obtained from standard 2D model at 100 ng/ml GLP-1). There were significant difference (p<0.05) between 3D static and standard 2D model at control (8 mM glucose medium) and 0.1 ng/ml GLP-1 in the presence of 8 mM glucose. By contrast islets cultured in three models showed the same pattern to response to tolbutamide stimulation in a dose-dependent monophasic manner and peaked at 20 µM (Figure 4). However, at the whole range of dose applied in the experiment, the highest insulin release response to tolbutamide stimulation was seen from Islet TF model whereas lowest were from standard 2D model while 3D static was in between. Significant difference between Islet TF and 3D static, Islet TF and standard 2D models were obtained p<0.001 at control (medium containing 8 mM glucose) (17.1±0.4 ng Insulin/mg cellular protein/min from Islet TF, 4.0±0.7 from 3D static and 1.5±0.1 from standard 2D), and p<0.05 at 20 µM tolbutamide in the presence of 8 mM glucose (23.1±1.0 ng Insulin/mg cellular protein/min from Islet TF, 8.4±3.2 from 3D static and 3.3±1.2 from standard 2D model), and 80 µM tolbutamide in the presence of 8 mM glucose (19.2±1.7 ng Insulin/mg cellular protein/min from Islet TF, 4.0±1.2 from 3D static and 1.8±0.3 from standard 2D model).

Reactivation of tolbutamide stimulation of insulin release from Islet TF and standard 2D model in the presence of 8 mM glucose.

**Figure 5:**
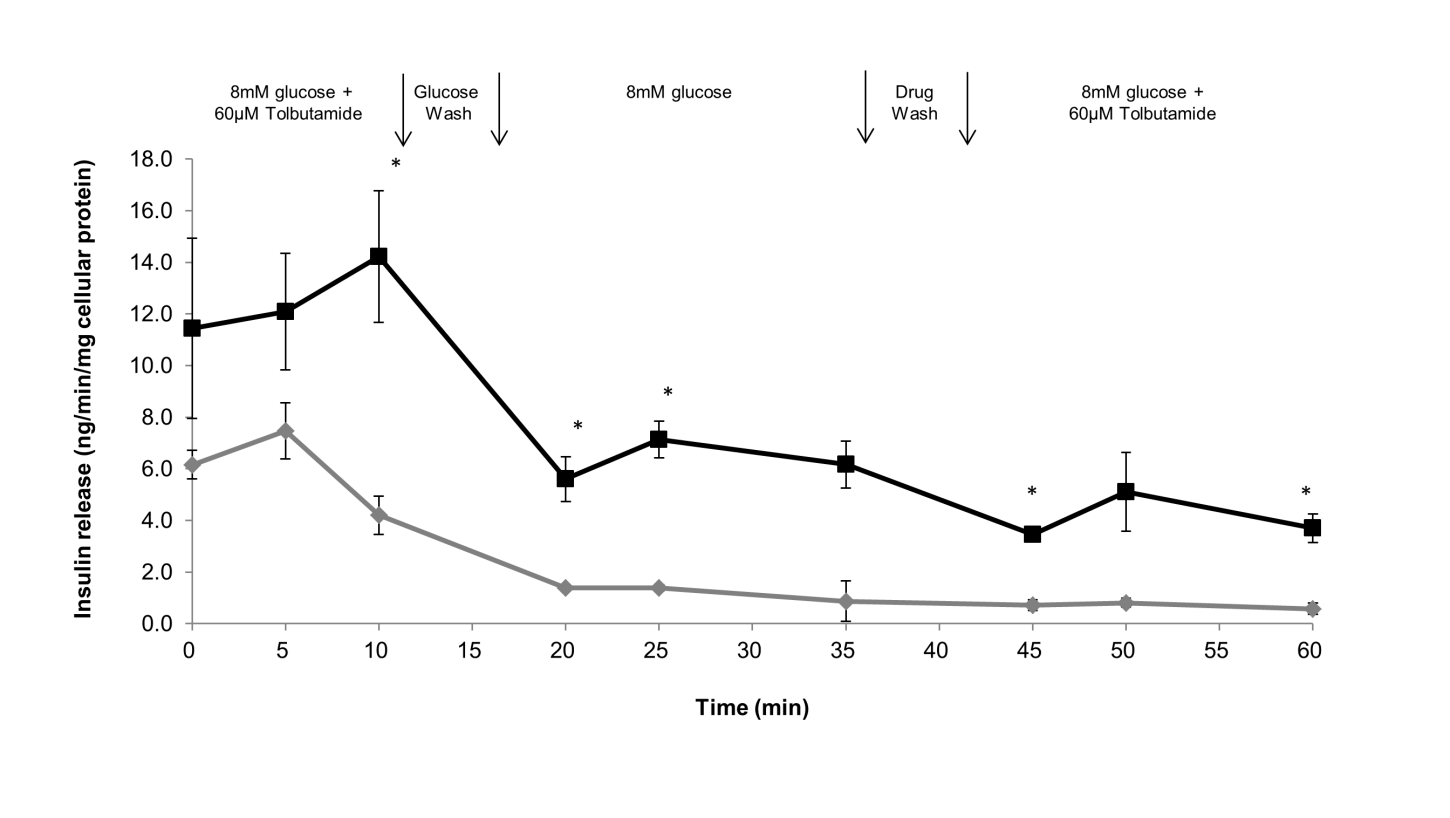
Reactivation of tolbutamide stimulation of insulin release detected from different islet models. Reactivation of tolbutamide stimulation of insulin release from islets cultured in Islet TF (black line) and standard 2D suspension (grey line) in the presence of 8 mM glucose was studied. After an initial equilibrium period of 15 min (min -15 to 0) in medium containing 8 mM glucose, tolbutamide (60 µM) was added to the medium in the present of 8 mM glucose at min 0, and was withdrawn at min 10, washed with medium containing 8 mM glucose and re-equilibrium for 20 min and reintroduced tolbutamide (60 µM) in the present of 8 mM glucose at min 35 and last 25 min. Values are means ± SEM of 3 independent experiments, each performed in duplicate. Statistical significance of difference between Islet TF and standard 2D models was calculated by Student’s T-test, * p<0.05.

To assess the sensitivity of Islet TF to response the cycle of drug introduction, we designed the experiment that mimics clinical drug administration process as follow: drug administration, withdrawal and reintroduction. Briefly islets were pre-cultured with 8 mM glucose for 15 min and then exposed to drug for 10 min, and then cultured in drug-free medium for 15 min, and then re-exposed to drug for 20 min. Figure 5 shown that islets cultured in both Islet TF and standard 2D models maximally responded to drug exposure in 10 min after first addition of the tolbutamide and then diminished with time. After the cycle of withdrawing the drug and reintroducing the drug, islets TF showed the second round of response to drug similar to that following the first treatment, whereas islets in standard 2D model lost their ability further responding to the drug. Although the second response to the drug was weak, the readmission stimulated insulin secretion was obtained only in Islet TF suggested that islets cultured in Islet TF relatively retained their sensitivity in insulin release potential response to the drug compared to standard 2D model.

### Effect of GLP-1 on glucose-stimulated insulin release in Islet TF model

**Figure 6:**
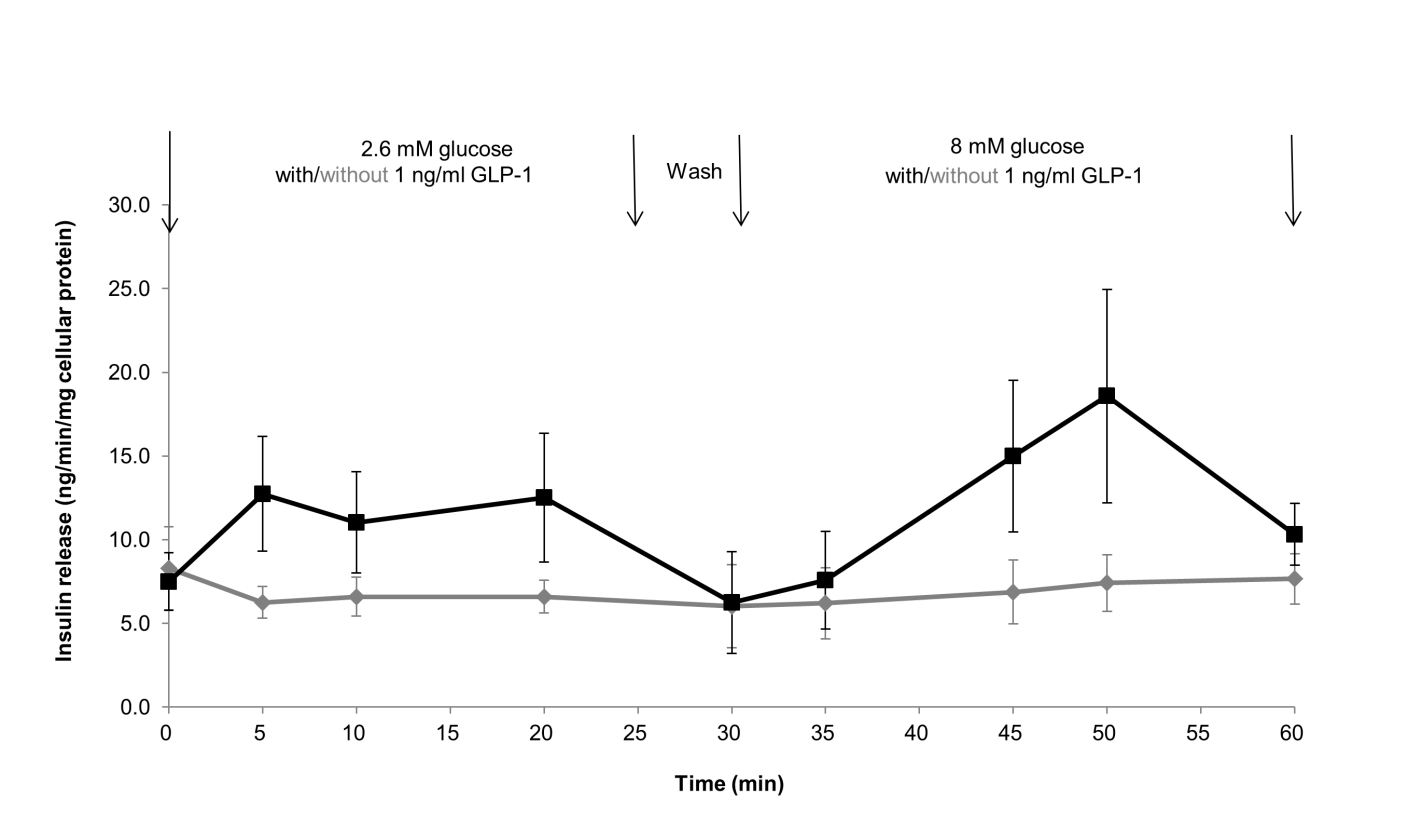
Kinetics of combination of GLP-1 and glucose stimulated insulin release from Islet TF model. Effect of GLP-1 on the kinetics of a subsequent glucose-stimulated insulin release from rat islets cultured in Islet TF model was studied. Islets cultured in Islet TF were stimulated with 1 ng/ml GLP-1 from min 0 to 60 combined with medium glucose concentration increasing from 2.6 mM to 8 mM from min 30 to 60 (black line). The control release of insulin by islets cultured in Islet TF with glucose concentration increasing alone is also shown (grey line). Values are means ± SEM of 3 independent experiments, each performed in duplicate.

Glucose concentration is one of the most important factors to regulate insulin release. The selection of glucose and drug concentration and their combination are the vital parameters for *in vitro* diabetic drug efficacy testing. Here we investigated insulin release response to one dose of GLP-1 (1 ng/ml) in two glucose concentration (2.6 mM and 8 mM) using Islet TF model. The control biphasic release of insulin produced by an increase of the glucose concentration from 2.6 to 8 mM was shown in Figure 6. GLP-1 at 1 ng/ml steadily increased the rate of insulin secretion in presence of 2.6 mM glucose and peaked at the level of 12.7±3.4 and 12.5±3.8 ng/min/mg cellular protein (two-fold increase normalized to control) at 5 and 20 min post-drug exposure respectively. The second peak appeared at the level of 18.6±6.4 ng/min/mg cellular protein (two and half-fold increase normalized to control) at 50 min after exposure of GLP-1 in presence of 8 mM glucose. As the concentration of GLP-1 was the same in two phases, the markedly elevated insulin release at phase two was solely due to combination of GLP-1 addition and presence of 8 mM glucose.

## Discussion

*In vitro* 3D cell culture models may provide more accurate prediction of drug toxicity and efficacy by enabling cells to be tested in an environment that more closely mimics the *in vivo* state. Compared to conventional suspension culture, *in vitro* viability and functions of islets were improved when they were cultured in three-dimensional collagen-based matrix [9,10,25–27] and hydrogel [28]. Furthermore encapsulation of islets in alginate has been proposed to protect islets from immune-mediated destruction [29,30], and improved islet cell survival *in vitro* and enhance metabolic function *in vivo* following transplantation [6,31–33]. However, a problem associated with static culture of islets in 3D matrices is the diffusion of nutrients and waste products across the matrix *in vitro* [34]. This could be overcome by utilising the dynamic culture to enhance diffusional limitation and to provide a better control of local micro-environment, e.g. better gas exchange, higher mass transfer, more physiological local solute concentrations and shear stress [35,36]. Among the dynamic culture system available on the market, TissueFlex^®^, a multichannel single direction perfusion culture system, is particularly suitable for long-term culture of small-scale 3D hydrogel-based cell/tissue models [16,21]. In this study, we demonstrate that 3D encapsulation of islet is essential but perfusion culture is the key to maintain *in vitro* islet viability and function in culture. Islets embedded in alginate maintained high islet viability in 7 days culture under both static and perfusion culture conditions (Figure 1). Islet function, however, was significantly influenced by culture conditions. Perfusion culture of 3D islets in TissueFlex^®^ (Islet TF) dramatically improved islet functions compared to 3D static and conventional 2D at magnitude of 5 to 9 folds on day1 and about 10 folds on day3 (Figure 2A & B). Dynamic culture has been reported to improve cell viability, proliferation and functions in many cell types including hepatocytes [11,17], osteoblasts [15,35], chondrocytes [36], and stem cells [21,37]. However, this is the first time we demonstrate that perfusion culture of islets which are highly metabolically active mini-organelles in 3D successfully retain *in vitro* islet viability and functions at a great level for 7 days.

As the features of islets such as high metabolically active, sensitive to glucose concentration, medium components are vitally important for *in vitro* islet culture, particularly when performed under 3D perfusion culture condition. Some studies showed that oxygen uptake, glucose oxidation and insulin synthesis became depressed when islets were cultured in low glucose containing media [38] whereas exposure to a high glucose concentration resulted in a decrease of insulin content [39]. Furthermore, following a glucose challenge, islets cultured in media with higher glucose concentrations (e.g. 11 mM and 28 mM glucose) were unable to respond to an increase in glucose concentration further because their basal release was already elevated [40]. Increasing evidences showed that culture medium containing 5.5 mM glucose was more suited for conducting such tests [6,22,34]. Among five commercial available media including RPMI 1640, CMRL 1066, MEM, DMEM, DMEM-F12, we found that the viability and integrity of islets in alginate beads were best maintained in perfusion culture using DMEM-F12 medium containing 5 mM glucose, while no differences were observed with any of these media in standard 2D format (data not shown).

Insulin is synthesized in the pancreas within the beta cells of the islets of Langerhans and stored inside mature granules and released from the cell into the circulation when any of several stimuli are detected. The primary release of insulin from beta cells is rapidly triggered in response to increased blood glucose levels while the second phase is a sustained, slow release of newly formed vesicles triggered independently of sugar. This is a dynamic biological process from insulin synthesis to release. The conventional perifusion technique is an inspective method utilized to study the dynamics of insulin release in a very short period (less than 3 hours) [41,42], but TissueFlex^®^ is better suited for conducting tests of cell/tissue functions and their dependence on drugs and chemicals. TissueFlex^®^ is designed for conducting parallel 3D perfused cell/tissue culture. The perfusion can maintain the chemostatic environment around the culture cells and tissue, enabling the directly link between extracellular conditions to cell/tissue function. TissueFlex^®^ enables precisely control the chemostatic environment in a mid-throughput and parallel manner during the drug dosing period by constantly supplying fresh media, which may contain drugs at preset doses, and removing waste products through pumping system that mimics the circulatory system in the body. Here we demonstrate that TissueFlex^®^ can be used to develop *in vitro* islet models and indeed functional insulin release response to chemical stimuli and dose dependence could be conveniently performed directly using the TissueFlex^®^ system.

As proof of concept, the dynamics of insulin release response to two anti-diabetic drugs were studies using the Islet TF model. Tolbutamide and GLP-1 are two typical drugs used to treat type II diabetes with different mechanisms. Tolbutamide is a first generation potassium channel blocker, sulfonylurea oral hypoglycaemic drug to treat Type II diabetes by stimulating the secretion of insulin by the pancreas [43,44]. GLP-1 is a potent antihyperglycemic hormone by inducing glucose-dependent stimulation of insulin secretion [45]. The potency of both GLP-1 and tolbutamide was dependent on the glucose concentration [23,24]. Our preliminary study indicated that islets rapidly release insulin response to drug stimulation preferably in the presence of 8 mM glucose (data not shown). Under such condition, Islet TF model tests revealed that an increased insulin release was triggered by the presence of the drugs and peaked at 1 ng/ml for GLP-1 and 20 µM for tolbutamide (Figures 3 & 4). The elevated insulin release from Islet TF, as the most important function indicator, is of vital importance for improving sensitivity in drug test and for determining the suitable dosages.

The actual drug administration in clinics can be simulated using Islet TF model by studying reactivation of tolbutamide stimulation of insulin release and GLP-1 on the kinetics of a subsequent glucose-stimulated insulin release. Dynamic insulin release response to tolbutamide addition, withdrawn and re-introduction was captured in Islet TF. Such behavior was not observed in conventional 2D islet culture where only first tolbutamide stimulation was detected. With the Islet TF model, during both the first and second tolbutamide additional period, insulin release reached maximum in 10 min post-drugging (10 min at first drugging period and 50 min at second drugging period) and then diminished with time (Figure 5). This finding differed from other studies stating that the releasing effect of the drug was maximal immediately after addition of tolbutamide [24]. Chicheportiche and Reach reported the delay response in static 3D alginate culture [46]. However, it seems unlikely that a delayed insulin release occurrence due to diffusion of protein through alginate beads in TissueFlex system as the system provides forced proactive flow rather than passive diffusion dominating in static culture. Islet TF displayed a high sensitivity in response to drugs, as such, it might imply that our data revealed a delayed of insulin release when adding tolbutamide which was not observed in the previous study [24]. Henquin JC reported that insulin release could be sufficiently stimulated if only tolbutamide combining sulphonylurea were re-introduced in the medium [24], which explained why only a weak peak was observed after second addition of tolbutamide at 50 min in our experiment.

The insulinotropic action of GLP-1 is glucose dependent [47]. The biphasic pattern of *in vitro* GLP-1 potentiation of glucose-stimulated insulin secretion detected in Islet TF was in agreement with that observed *in vivo* [47]. The second biphasic pattern was expected when adding GLP-1 in presence of 8 mM glucose at phase two. However, only monophasic pattern peaked at 50 min was detected. The reason why this second peak was not observed is worth further investigating, for example, by conducting longer experiments to confirm whether any delaying in appearance.

## Conclusion

In this study we demonstrate for the first time that perfused 3D culture of islets (the Islet TF model) can maintain the *in vitro* islet viability, and more importantly, the elevated functionality in terms of insulin release and dynamic responses over a 7-day culture period. The Islet TF displays a high sensitivity in responding to drugs and drug dosages over conventional 2D model. Actual drug administration in clinics could be simulated using the developed Islet TF model, and the patterns of insulin release response to the tested drugs were in agreement with the data obtained *in vivo*. Islet TF could be a more predictive *in vitro* model for routine short- and long-term anti-diabetic drug efficacy testing.
